# A Trade-Off Analysis between Sensor Quality and Data Intervals for Prognostics Performance

**DOI:** 10.3390/s22197220

**Published:** 2022-09-23

**Authors:** Hyung Jun Park, Nam Ho Kim, Joo-Ho Choi

**Affiliations:** 1 Department of Smart Drone Convergence Engineering, Korea Aerospace University, Goyang-si 10540, Korea; 2 Department of Mechanical and Aerospace Engineering, University of Florida, Gainesville, FL 32611, USA; 3 School of Aerospace & Mechanical Engineering, Korea Aerospace University, Goyang-si 10540, Korea

**Keywords:** prognostics, sensor quality, data interval, performance metric, accelerometer, microphone

## Abstract

In safety-critical systems such as industrial plants or aircraft, failure occurs inevitably during operation, and it is important to prevent it in order to maintain high availability. To reduce this risk, a lot of efforts are directed from developing sensing technologies to failure prognosis algorithms to enable predictive maintenance. The success of effective and reliable predictive maintenance not only relies on robust prognosis algorithms but also on the selection of sensors or data acquisition strategy. However, there are not many in-depth studies on a trade-off between sensor quality and data storage in the view of prognosis performance. The information about (1) how often data should be measured and (2) how good sensor quality should be for reliable failure prediction can be highly impactful for practitioners. In this paper, the authors evaluate the efficacy of the two factors in terms of remaining useful life (RUL) prediction accuracy and its uncertainty. In addition, since knowing true degradation information is almost impossible in practice, the authors validated the use of the prognosis metric without requiring the true degradation information. A numerical case study is conducted to identify the relationship between sensor quality and data storage. Then, real bearing run-to-failure (RTF) datasets acquired from accelerometer (contact type) and microphone (non-contact type) sensors are evaluated based on the prognosis performance metric and compared in terms of the sensors’ cost-effectiveness for predictive maintenance.

## 1. Introduction

In many engineering systems, prognostics and health management (PHM) technologies draw extensive attention as a means to enable predictive maintenance. The steps of PHM usually begin with data acquisition from sensors, where different kinds of condition-monitoring data are captured and stored from various sensors that may contain health condition information of the equipment in concern [1]. From the measured data, a health indicator (HI) can be constructed to represent the health condition using signal-processing techniques, feature extraction, and/or machine learning methods. Afterward, a remaining useful life (RUL) until failure is predicted based on the degradation trend of the HI using various prognostics algorithms.

While a lot of research has focused on the techniques for the latter two steps, fewer investigations have been made to the first step, which includes data measurement management and the performance evaluation of various sensor technologies. It is evident that acquiring many measurement data from high-quality sensors is an ideal situation for building a successful PHM function. However, there are several practical issues to be considered. First, more measurement data is directly related to higher data storage and processing costs. In a similar context, high-quality sensors are known to be more effective than low-quality ones, but they are expensive to purchase and their operating costs are high [2,3]. Therefore, it would be beneficial to evaluate the trade-off between the sensor quality and the amount of data storage to obtain reliable prognostics results.

General methods of sensor evaluation and selection for prognostics are based on the trend of measurement itself or the extracted HI. Liu et al. [4] proposed a permutation entropy-based strategy to quantitatively select sensors that reflect the monotonic trend of degradation to perform aircraft engine health prognosis. Moreover, they further used an improved 2! permutation entropy method to optimize the number of selected sensors for prognostics [5]. Zhang et al. [6] and Coble et al. [7] developed a sensor selection metric that considers the trend consistency of sensor data among different systems and validated it with engine simulation datasets. Kim et al. [8] proposed a latent linear model for HI construction and used a lasso method to select useful sensor signals for the developed model. Yu et al. [9] selected sensors with a monotonic trend prior to developing a single HI, and then further evaluated the grey correlation analysis between the HI and each of the selected sensors to decide the optimal sensor subset. Summing up the existing literature, the main focus is found on evaluating the sensor performance in the prognostics using the metrics of monotonicity, correlation, and robustness. However, it appears that there is a lack of study in the context of two factors, namely the sensor quality and amount of data, since they consequently affect the accuracy and the uncertainty of RUL prediction. 

Few studies have considered the sensor quality in this aspect. Dawn et al. [10] evaluated crack growth prediction and model parameters estimation performance using Bayesian inference when the observed data have noise and bias. In the results, the authors were able to predict a crack with accuracy only if there were many data with large noise. Camci et al. [11] focused on developing a guideline for practitioners on various types of sensors used for railway turnout system monitoring. The RUL performance of each sensor is evaluated and the additional cost factors of sensors are considered to make an economic justification for the optimal sensor selection. However, the above studies aimed at the sensor quality related to noise interference, but reducing the number of data to monitor is not considered. More recently, Zhu et al. [12] proposed an active querying-based training data selection mechanism in a Deep Learning (DL)-based prognosis to select the most informative sample from unlabeled data. Kim et al. [13] proposed proper measurement scheduling while maintaining the prediction uncertainty. These studies provide a revealing insight on optimal data management but studies relating to sensor quality are still limited. Moreover, the metrics to evaluate prognosis performance in the studies are based on true degradation information [14,15], such as the true end of life (EOL), the true degradation curve, or the historical run-to-fail data, which is challenging to assess in practice.

Motivated by the above issues, this paper evaluates the sensor performance in view of the amount of data by varying the monitoring interval and the sensor quality by varying the level of noise. In addition, the prognosis performance is evaluated using a metric without true degradation information. Eventually, we want to answer the following two questions: (a) how good sensors should be employed and (b) how often data should be measured. The relationship between the sensor quality and the number of data is identified through numerical study and real run-to-failure (RTF) datasets acquired from two different type of sensors in a test rig, namely, the accelerometer [16] and microphone, in which the former is the most common and inexpensive contact sensor, while the latter is a non-contact acoustic sensor with higher cost and quality. The microphone is recently drawing attention as an alternative means due to its advantage of low interference with external noises within the system [17]. 

The rest of the paper is organized as follows. Section 2 presents the proposed methodology and theoretical background for algorithms. Section 3 addresses the correlation analysis between two different metrics and establishes a relationship between the two factors based on the numerical study. Then, Section 4 presents the application with the bearing RTF datasets of the designed experiment. Lastly, conclusions and potential future works are presented in Section 5.

## 2. Methodology

**Figure 1:**
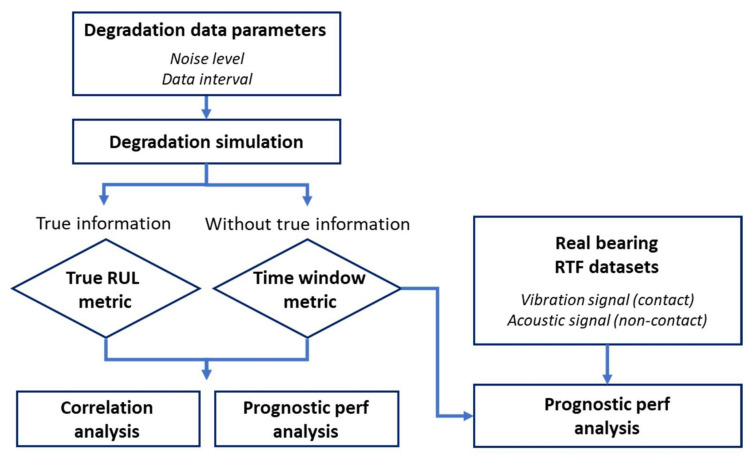
The overall framework of the sensor evaluation.

An overall framework of the study is described in Figure 1. In the beginning, sensor performance is analyzed by simulation study as shown in the left part of the figure. First, the two parameters related to the sensors, namely the level of noise and data intervals (amount of data), are varied through numerical simulation to generate various datasets of degradation. Then, two different metrics are introduced to evaluate the prognostic performance. One is the true RUL metric evaluated based on the true information such as the true EOL and the degradation curve, while the other is the time window metric evaluated based only on the subsequent measurements. A correlation analysis between the two is conducted to validate the use of the time window metric, and the relationship between the two parameters is identified in view of the prognostic performance. Finally, the bearing RTF experiment is conducted with two different types of sensors, an accelerometer (contact) and a microphone (non-contact), as shown in the right part of the figure. Then, the time window metric is applied to compare the performance of the two sensors.

### 2.1. Degradation Simulation

In this section, virtual degradation datasets are generated by numerical simulation with different levels of noise and data intervals. The degradation function is assumed to follow an exponential function since various components such as the battery and bearing degradation are widely known to be degraded exponentially [18,19,20]. Thus, the degradation model is given as

(1) $$y=exp\left(bt\right)$$

where t is the cycle, b is an exponential parameter set with a constant value of 0.02, and y is degrading HI. The degradation data were generated for 100 cycles using Equation (1). Then, the following measurement model was used to add random noise to the degradation data:

(2) $$z=y+\varepsilon ,\;\;\varepsilon \sim U\left(-Lv.noise,\;Lv.noise\right)$$

where ε is uniformly distributed with a certain level of noise. In this study, the degradation data was generated with intervals (Δt) varying from 1 cycle to 8 cycles and four different noise levels from 0.2 with the increment 0.1. The values for set data parameters are summarized in Table 1.

**Figure 2:**
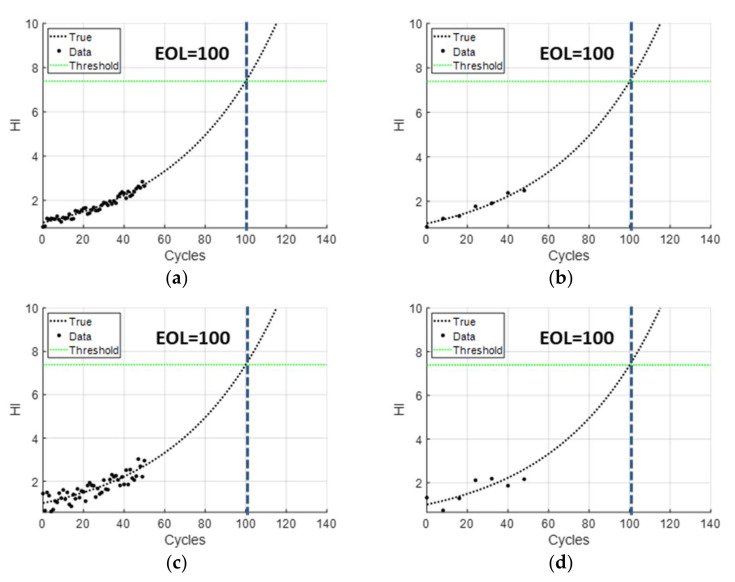
Simulation degradation datasets: (**a**) low noise and large data; (**b**) low noise and small data; (**c**) large noise and large data; (**d**) large noise and small data.

Considering four noise levels and four data intervals, a total of 16 cases were studied. Figure 2a shows an example of the dataset with a small level of noise (0.2) and a small data interval (Δt=1). The black dots and dashed curve refer to measured data until 50 cycles and the true degradation curve until the EOL, respectively. The green horizontal line is the threshold at EOL, which is used to predict RUL. Figure 2b shows the case of a small level of noise (0.2) and a large data interval (Δt=8). Figure 2c,d are cases under a large level of noise (0.5) with a small data interval © and a large data interval (d), respectively. Since the randomness in the noise can lead to different prediction performances even under the same parameter, 50 datasets were randomly generated under the same condition and used to assess the performance of prediction.

### 2.2. Regularized Particle Filter

The particle filter (PF) algorithm, also known as the Sequential Monte Carlo method, is a widely used prognostic approach in many engineering problems such as Lithium-ion batteries, induction motors, and PEM fuel cells [21,22,23]. PF recursively estimates and updates the probability density function (pdf) of the unknown model parameters or states of interest in the form of particles, based on the following Bayes’ theorem:

(3) $$p\left(\theta \left|z\right.\right)\propto L\left(z\left|\theta \right.\right)p\left(\theta \right)$$

where θ is a vector of unknown parameters, z is a vector of measurements, $L\left(z\left|\theta \right.\right)$ is the likelihood of obtaining measurement z given parameter θ, $p\left(\theta \right)$ is the prior pdf of θ, and $p\left(\theta \left|z\right.\right)$ is the posterior pdf of θ conditional on z. The standard PF consists of state transition function f to predict the evolution of the state and measurement function h  as follows:

(4) $$x_{k}=f\left(x_{k-1},b_{k},v_{k}\right)=exp\left(b_{k}dt\right)x_{k-1}$$

(5) $$z_{k}=h\left(x_{k},n_{k}\right)=x_{k}$$

where k is the time step index, $x_{k}$ is the state, $b_{k}$ is the vector of model parameters, $z_{k}$ is the measurement data, and $v_{k}$ and $n_{k}$ are the process and measurement noises, respectively. In practical applications, the process noise can be ignored since it can be included in the model parameter’s uncertainty.

In this study, the exponential function was used for the transition function and the process noise was ignored since it can be handled through the uncertainty in the model parameters. For measurement, it was assumed that $z_{k}$ was the same as degradation data including measurement noise from a Gaussian distribution, $n_{k}\sim N\left(0,\sigma_{k}\right)$, where $\sigma_{k}$ is the unknown parameter of the standard deviation of the noise. Thus, the total unknown parameters to be estimated were $\theta ={\left[x,b,\sigma \right]}^{T}$.

The process of the PF is composed of three steps at each iteration. First, in the prediction step, particles in the previous time step are propagated through the transition function to form particles at the current time, which is the prior pdf $p\left(\theta \right)$ at the current time. Then, in the updating step, the likelihood of measurement data $L\left(z\left|\theta \right.\right)$ that represents each particle’s weight is calculated. As a new measurement is used, the weight of each particle is adjusted. A higher weight is assigned to the particles that have a higher similarity with the measurement. Finally, in the resampling step, the particles are rearranged based on the likelihood, such that they are duplicated or eliminated depending on the weight of the particles by using the inverse cumulative distribution function (CDF) method [24,25]. The resampled particles, which are the posterior distribution at the current time are then used as the initial distribution for the next step prediction. More information about PF can be found in An et al. [26].

Due to the resampling process, PF-based prognosis often suffers the problem of particle impoverishment since the samples are drawn from a discrete distribution rather than a continuous one. Consequently, after several iterations, the particles with small weights are discarded and the particles with high weights are duplicated multiple times, which gives a poor representation of the posterior distribution. To resolve this issue, a regularized Particle filter (RPF), which is a modified version of PF, was used in the resampling step. Kernels were generated at particle points and summed to generate the kernel density estimate in RPF to have the advantage of approximating the weighted particles in continuous distributions [27].

### 2.3. Prognostic Performance Metric

**Figure 3:**
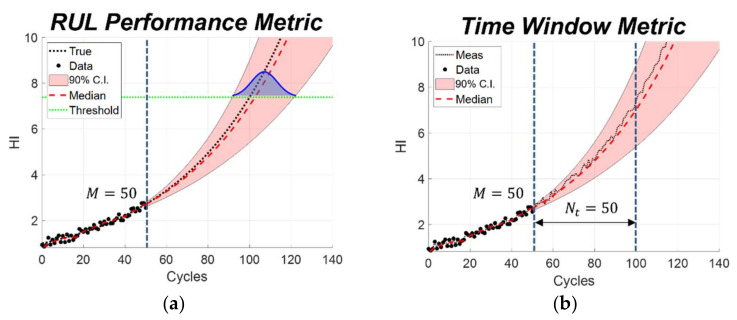
Illustration of two different prognostic performance strategies. (**a**) RUL performance metric; (**b**) Time window metric.

After predicting the future degradation using the RPF algorithm, two different prognostic performance metrics were calculated: the RUL performance metric and the time window metric. To compare the performance of both metrics, two measures were considered: the measure of the prediction accuracy and the measure of the uncertainty associated with the prediction. The schematic illustration of each metric calculation is addressed in Figure 3.

The RUL performance metric was calculated based on the result shown in Figure 3a. The black dots and black dashed line represent the measured data until the current cycle (M=50 cycles) and the true degradation curve, respectively. Measurement data until M cycles were used for estimating the distribution of model parameters. The red dashed line denotes the prediction median at the future cycles, whose 90% confidence intervals are shown in the shaded area. The horizontal green dotted line is the threshold until failure. Since the true degradation curve crosses this line at 100 cycles, the EOL is 100 cycles. When the predicted state reaches the threshold, we can obtain the distribution of EOL cycles. The distribution of RUL can be obtained by subtracting this distribution from the current cycle, which is cycle 50. From the predicted RUL distribution, the accuracy measure can be calculated by Equation (6), which is an absolute error between the median of the predicted RUL and that of the true RUL. Besides prediction accuracy, the level of uncertainty associated with the prediction is also an important factor to assess the prognostic performance from a conservative point of view. Therefore, the level of uncertainty was considered as normalized C.I., which is defined as $RUL_{CI}$ in Equation (7), where $RUL_{pred,5\mathrm{th}}$ and $RUL_{pred,95\mathrm{th}}$ are the 5th and 95th percentiles of the predicted RUL, respectively. The performance of the prognostic algorithm is considered to be good when both metrics are close to zero.

(6) $$RUL_{error}=\left|RUL_{pred,median}-RUL_{true}\right|$$

(7) $$RUL_{CI}=\frac{RUL_{pred,95\mathrm{th}}-RUL_{pred,5\mathrm{th}}}{RUL_{true}}\;$$

The time window metric is different from the RUL metric as it directly uses the measurements in a certain time window without the information of the true degradation [28]. The strategy is shown in Figure 3b, where the black dashed line is not the true degradation curve, but measurement data in the future. Since the true degradation value is not available, the predictions can be compared with the subsequent measured data only. Thus, the prediction accuracy and the level of uncertainty are assessed over a time window, which is the range between the two vertical dashed lines in Figure 3b. The width of the time window ($N_{t}$) is set based on how much early prediction is required for maintenance scheduling. In the simulation study, the window was set to 50 cycles. The normalized mean square discrepancy (*NMSD*) was introduced to assess the prediction accuracy using the following equation:

(8) $$NMSD=\frac{\frac{1}{N_{t}}\sum_{i=1}^{N_{t}}{\left(z_{M+i}-{\hat{x}}^{m}{}_{M+i}\right)}^{2}}{\max\left(z_{M+\left(1:N_{t}\right)}\right)-\min\left(z_{M+\left(1:N_{t}\right)}\right)}$$

where M is the prediction start cycle, z is the measured data in the time window, and ${\hat{x}}^{m}$ is the predicted median of the degradation state by the prognostic algorithm. *NMSD* measures the relative difference between subsequent measurements and the mean predictions. The performance of the prognostic algorithm is considered to be good when NMSD is close to zero. 

For the uncertainty measure, two indices were considered together. The first index $E_{1}$ measures the relative width of the 90% C.I. with respect to the predicted median value for each cycle and averages over the time window, as

(9) $$E_{1}=\frac{1}{N_{t}}\overset{N_{t}}{\underset{i=1}{\sum }}\frac{\left({\hat{x}}^{u}{}_{M+i}-{\hat{x}}^{l}{}_{M+i}\right)}{{\hat{x}}^{m}{}_{M+i}}$$

where ${\hat{x}}^{l}$ and ${\hat{x}}^{u}$ represent the 5th and 95th percentiles of the prediction, respectively. A smaller $E_{1}$ indicates a narrower C.I. over the prediction and lower prediction uncertainty. The second index $E_{2}$ measures whether the C.I. of the prediction covers the data and how wide the C.I. needs to be to cover the data at each prediction point. Specifically, at each prediction cycle in the time window, the minimal α% C.I. that can cover the data is calculated. The discrete values of α are increased from 90 to 99 with one increment, and $E_{2}$ is calculated as 1−0.01α, which varies between 0 and 0.9. Since a smaller α indicates a more reliable prediction, a larger value is assigned for $E_{2}$. If the highest α=99 cannot cover the data, $E_{2}$ becomes zero.

(10) $$E_{2}=\left\{\begin{array}{cc} \frac{1}{N_{t}}\sum_{i=1}^{N_{t}}\left(1-0.01\alpha_{M+i}\right) & \;,\alpha =90,\;91,\;\ldots \;,\;99\;\;\;\;\;\;\;\;\;\;\;\;\;\;\\ 0 & ,\;\mathrm{if}\;\mathrm{required}\;\alpha \;\mathrm{exceeds}\;99 \end{array}\right.$$

The above two indices evaluated the prognostic performance from the perspective of uncertainty. A small $E_{1}$ and a high $E_{2}$ represent good prediction. Thus, the nonlinear combination of $E_{1}$ and $E_{2}$, $EI=E_{2}/E_{1}$ was defined as an index to assess uncertainty performance for the time window metric.

## 3. Numerical Case Study

In this section, we attempt to evaluate the correlation between the RUL performance metric and the time window metric. Since the prediction performance of the prognostic algorithm can differ by the randomness of noise even under the same noise level, the correlation was analyzed using multiple randomly simulated datasets (50 datasets). If the two metrics have a high correlation, the time window metric can be used with confidence to assess the prediction performance using the measurement data only without true degradation information.

**Figure 4:**
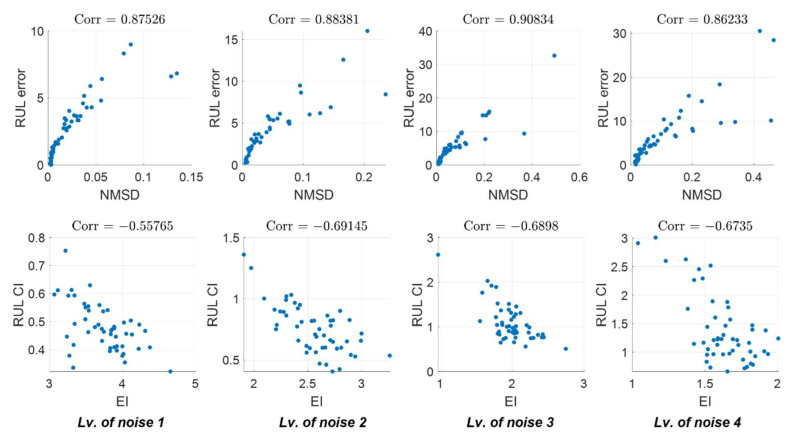
Correlation between two different metrics under varying noise.

The correlation was calculated between each component in metrics, the accuracy measure and uncertainty measure. The scatter plot and correlation coefficient value between the two metrics under different levels of noise are shown in Figure 4. The first-row figures present the scatter plot between $RUL_{error}$ and *NMSD*, and the second-row figures, between $RUL_{CI}$ and EI. The correlation between accuracy measures shows a very high correlation regardless of noise levels: the smaller the RUL error, the smaller the *NMSD* value. Though the uncertainty measure shows less correlation than the accuracy measure, the correlation coefficient shows over 0.5 regardless of noise. Note that they show a negative correlation since a higher EI means more reliable prediction, which corresponds to a smaller $RUL_{CI}$. As such, the time window metric is shown to have a high correlation with the true RUL performance and can be used as a prediction performance indicator when the true information is unavailable.

**Figure 5:**
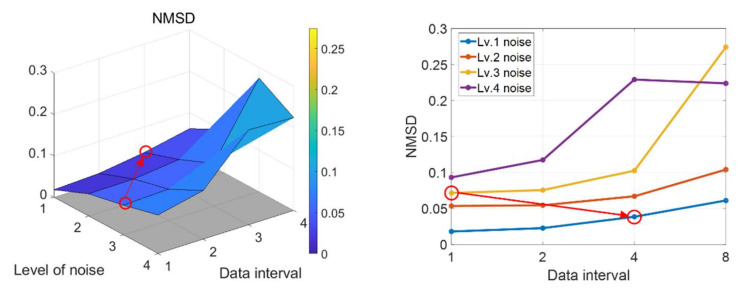
Prediction accuracy of datasets under various levels of noise with different data intervals.

Using the time window metrics, prediction accuracy was analyzed under various levels of noise and different data intervals until the current M cycles. A large interval means a small number of data for prognostic performance evaluation. The overall results of *NMSD* are presented in Figure 5. The left figure is the 3D plot of *NMSD* with respect to the two parameters (level of noise and data interval), while the right is a 2D projection with respect to the level of noise. The red circles and arrow show that Lv. 1 noise with Δt=4 has a better prediction accuracy (smaller *NMSD*) than Lv. 3 noise with Δt=1. Other comparisons between different level of noise show similar results. This can be interpreted as indicating that a high-quality sensor with a lower number of data outperforms a low-quality sensor with a higher number of data. Therefore, a trade-off between the sensor quality and data intervals can be possible.

## 4. Bearing Case Study

The bearing is one of the most critical components that leads to system failure, and numerous studies have been conducted to prevent its failure [29,30,31]. Among available sensors, the accelerometer is the most common and cost-effective sensor for health monitoring. However, it has drawbacks of structural interference with other signals due to its attachment to the system. Recently, non-contact acoustic sensors such as a microphone are drawing attention as an alternative since it is less affected by other signal interference [32,33]. To thoroughly evaluate the prognostic performance of the two sensors with different qualities, we conducted multiple run-to-fail (RTF) experiments using a testbed mounted with a commonly used accelerometer and a high-quality microphone. The sensor performance was evaluated using the time window metric from the previous section.

### 4.1. Experimental Setup

**Figure 6:**
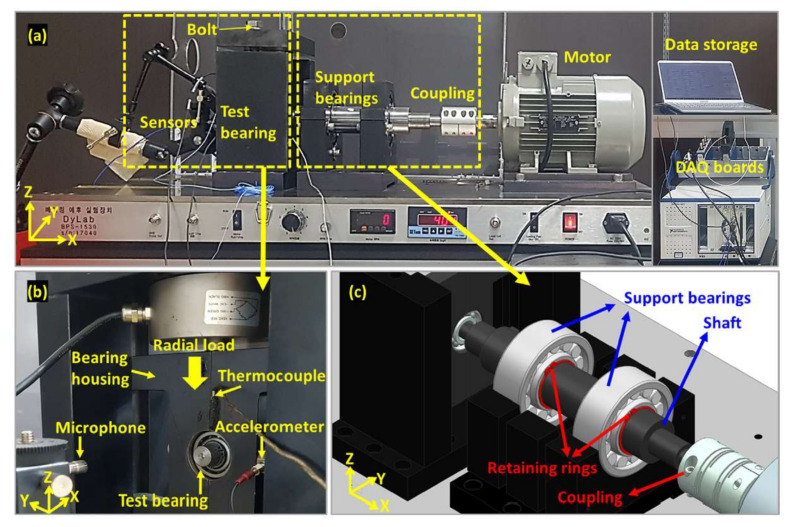
The bearing testbed (**a**) Front view of the bearing test rig (**b**) Close up view of test bearing and sensors (**c**) Close up view of support bearings and couplings.

Figure 6 shows the bearing test apparatus in which the RTF tests were performed. The apparatus consisted of sensors, test bearings, support bearings, motors, and DAQ boards. The sensors used in this study were an accelerometer (KS77C.100 by MMF), a microphone (PCB Piezotronics 378C01), and a thermocouple. The accelerometer was mounted to the right of the test bearing housing and the microphone was installed at a horizontal distance of 100 mm from the test bearing. The thermocouple was located 15 mm upward from the bearing. The cost of a microphone (1931 USD) was about 4 times higher than that of the accelerometer (452 USD). The DAQ boards consisted of an NI Pxle-4464 and an NI-9212, in which the former recorded acoustic and vibration signals at a sampling rate of 204.8 kHz, and the latter recorded the temperature at a 100 Hz sampling rate. The first 1 s of every 10 s was stored as one cycle using LabVIEW software.

The bearing was operated with 1700 rpm. A radial load was exerted by the mechanical fastening of bolts to the bearing located at the end of the shaft with the magnitude of 75~80% of the dynamic load rating of 7950 N to develop naturally growing defects. After a number of trials to ensure that the faults were fully developed over cycles while maintaining safety, the test was terminated when the acoustic pressure, acceleration, and temperature exceeded the thresholds of 9 Pascal, 18 $m/s^{2}$, and 80 °C, simultaneously. As result, three RTF test datasets were acquired by two different sensor signals having different levels of noise and patterns during degradation [17].

### 4.2. Sensor Performance Analysis

**Figure 7:**
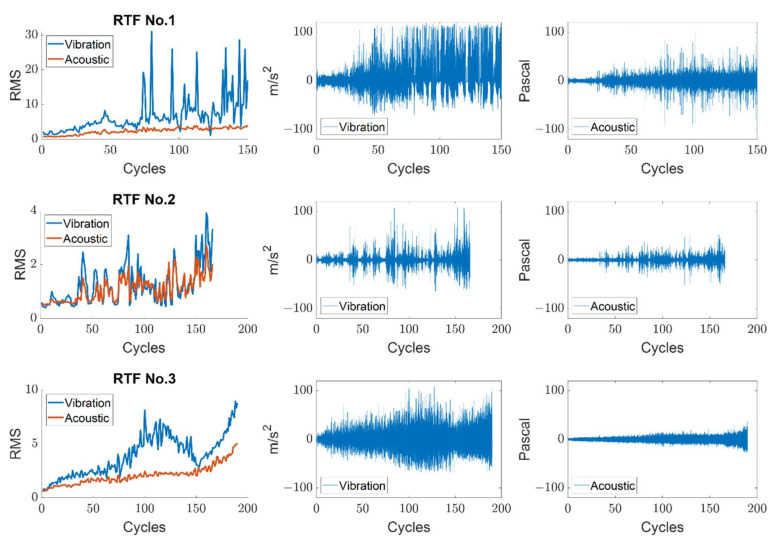
Comparison of RMS and raw vibration and acoustic signals for three run-to-fail datasets.

In general, the prognosis process requires extracting a proper health indicator (HI). In this study, as a traditional time-based statistical feature, the root mean square (RMS) of the signals is employed. Although there are many other HIs with better performance in view of accuracy and earlier prediction, the RMS is employed here because the main focus is on evaluating the prognostic performance of different-quality sensor data. The HIs and raw signals for three different RTF datasets are presented in Figure 7, where the lines with blue and red color are the HI from the vibration and acoustic signals, respectively. Corresponding raw signals are plotted next to the HI plot. It is noticeable that the overall vibration signals show high fluctuation and noise interference compared to the acoustic signals. For RTF No.3, for example, the vibration HI shows not only large noise but also high fluctuation on the degradation trend. As a result, it is observable that the signal acquired by the microphone was less influenced by the noise from the system such as friction between the shaft and the support bearing. 

**Figure 8:**
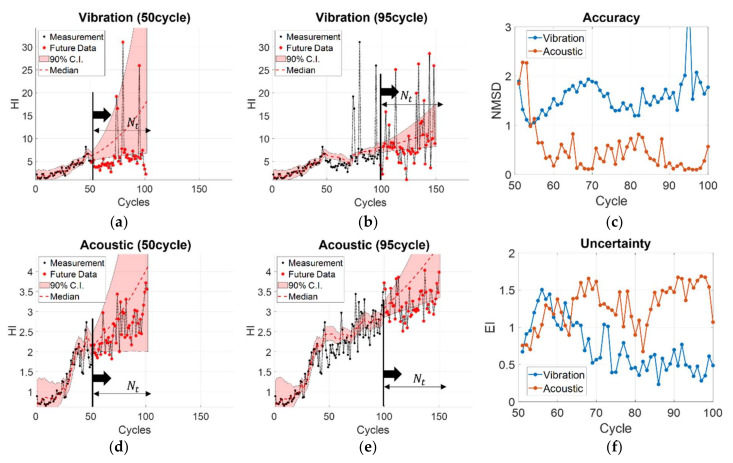
Prediction by the vibration sensor for RTF No.1 data at (**a**) 50cycle and (**b**) 95cycle; (**c**) *NMSD* calculated at each cycle from 50 to 95 cycles; Prediction by the acoustic sensor for RTF No.1 data at (**d**) 50cycle and (**e**) 95cycle; (**f**) EI calculated at each cycle from 50 to 95 cycles.

**Figure 9:**
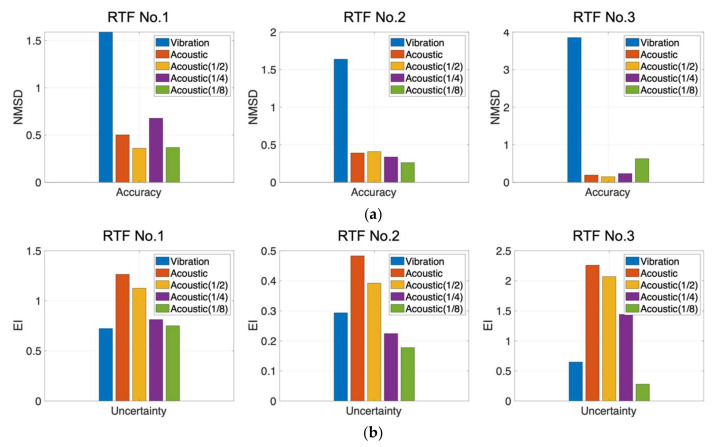
Average *NMSD* (**a**) and EI (**b**) of the vibration sensor, acoustic sensor, and acoustic sensor with different data intervals (1, 2, 4, and 8 cycles).

The prognosis performance for each sensor was calculated using the time window metric. The window size ($N_{t}$) was fixed at 50 cycles and initial prediction started from 50 cycles (M). Then, the performance metrics such as the accuracy (*NMSD*) and uncertainty (EI) were calculated for every cycle until failure. For example, Figure 8a,b shows the prediction results by the vibration sensor for RTF No.1 data when M is 50 and 95 cycles, respectively. Figure 8d,e are the prediction results by the acoustic sensor. The black and red dots denote the measurements until the current cycle and future cycles in the time window, respectively. The red dashed line and red shaded areas are the predicted median and 90% C.I. in the future. Figure 8c,f are the *NMSD* and EI calculated at each cycle from 50 to 95 cycles. The calculated *NMSD* and EI results obtained in Figure 8a,d are the first values for the vibration and acoustic sensors in Figure 8c,f, respectively. In order to compare the prognostic performance of the sensors, the values of *NMSD* and EI were averaged over all subsequent cycles, respectively. In the case of the acoustic sensor, the results were also obtained for different data intervals to examine its effect accordingly. The results are presented in Figure 9, in which the first- and second-row figures show the average *NMSD* and EI for the vibration and the acoustic sensors, respectively. In the figures, the first two bars are the results with data at every cycle, whereas the remaining three are the acoustic results with intervals of 2, 4, and 8 cycles (1/2, 1/4, 1/8 reduction in the number of data). The results indicate that, in terms of prediction accuracy, the *NMSD* of the acoustic sensor is smaller, hence, more accurate, than that of the vibration sensor in all the datasets, even when we use 1/8 amount of data for the prediction. In case of the uncertainty measure EI, the results of the acoustic sensor are higher than that of the vibration sensor when using the data with every cycle in all the datasets, which means that the reliability is higher. However, the value gets smaller for 1/4 data in RTF No. 2, and for 1/8 data in RTF No. 2 and 3, which means that the reliability becomes lower as the number of data are reduced. In summary, 1/2 (half) data are sufficient or even 1/4 (quarter) data are favorable for the acoustic sensor for prognosis in comparison with the results by the vibration sensor.

In order to achieve economic feasibility when employing an acoustic sensor, it may be necessary that the cost benefit of reduced data storage or inspection interval is evaluated as a trade-off to the higher cost of the sensor. This is, however, not addressed in this study. Nevertheless, the microphone, which has smaller noise, performs better in prediction even with four times fewer data than the accelerometer. Considering the cost occurrence due to false prediction and data storage for long-term monitoring, the microphone could be more cost-effective than implementing an accelerometer.

## 5. Conclusions

In this paper, a study toward a trade-off between sensor quality and data amount in the view of prognostic performance was addressed by numerical study and real application. First, the two parameters related to the level of noise and data intervals were considered to evaluate various datasets of degradation. Next, a correlation analysis between two different metrics was conducted to validate the use of the time window metric, which is only based on the subsequent measurements, without true run-to-failure data. Through the numerical study, the relationship between noise and data intervals was identified in view of the prognostic performance. In the real application study, two different sensor signals were obtained from a bearing degradation experiment. The results showed that the non-contact type microphone, which has a smaller level of noise, shows superior performance in overall prediction even with a quarter of the data than those of the accelerometer.

However, there still remains a number of critical challenges in order to build a guideline for practitioners. The cost of data storage or inspection needs to be considered in more detail to verify the economic feasibility of using high-cost sensors. The experiments of the two sensors were conducted in a controlled lab environment. Thus, other factors such as external noise and microphone placement distance should be considered additionally to evaluate the robustness of sensor performance. In addition, it is worth looking into the existing research gaps of the DL-based method in the perspective of data acquisition since it highly depends on the training datasets. Nevertheless, this study is a first step toward setting a guideline for the practitioners when establishing the data acquisition system for PHM.

## Figures and Tables

**Table 1:** Sensor data design parameters.

Lv. of noise	$\varepsilon \sim U\left(-Lv.noise,\;Lv.noise\right)\;Lv.noise=\left[0.2,\;0.3,\;0.4,\;0.5\right]$
Data interval	$\Delta t=t_{k}-t_{k-1},\;\Delta t=\left[1,\;2,\;4,\;8\right]$

## Data Availability

Not applicable.
